# Methyl 9-diethyl­amino-2,2-bis­(4-meth­oxy­phen­yl)-2*H*-benzo[*h*]chromene-5-carboxyl­ate

**DOI:** 10.1107/S160053681101049X

**Published:** 2011-03-26

**Authors:** Moon-Hwan Kim, Hee-Moon Park, Chong-Hyeak Kim

**Affiliations:** a Biomaterial Research Center, Korea Research Institute of Chemical Technology, PO Box 107, Yuseong, Daejeon 305-600, Republic of Korea; b Environment and Resources Research Center, Korea Research Institute of Chemical Technology, PO Box 107, Yuseong, Daejeon 305-600, Republic of Korea; c Center for Chemical Analysis, Korea Research Institute of Chemical Technology, PO Box 107, Yuseong, Daejeon 305-600, Republic of Korea

## Abstract

In the title compound, C_31_H_29_NO_5_, the methyl carboxyl­ate and dimethyl­amino groups on the naphtho­pyran group are almost coplanar with the naphtho­pyran ring system [r.m.s. deviations = 0.08 (2) and 0.161 (2) Å, respectively]. The dihedral angle between the methyl carboxyl­ate and dimethyl­amino groups is 4.9 (1)°. The pyran ring has an envelope conformation with the quaternary C atom out of plane by 0.4739 (13) Å. The meth­oxy­phenyl substituent forms a dihedral angle of 16.6 (1)° with the plane of the benzene ring, while the other meth­oxy­phenyl group is almost coplanar, making a dihedral angle of 1.4 (1)°.

## Related literature

For the synthesis and properties of organic photochromic and thermochromic dyes, see: Clarke *et al.* (2002 ▶); Gabbutt *et al.* (2003 ▶, 2004 ▶); Kim *et al.* (2010 ▶); Do *et al.* (2011 ▶). For their applications, see: Kumar *et al.* (1995 ▶); Gemert & Selvig (2000 ▶); Nelson *et al.* (2002 ▶); Crano & Guglielmetti (1999 ▶).
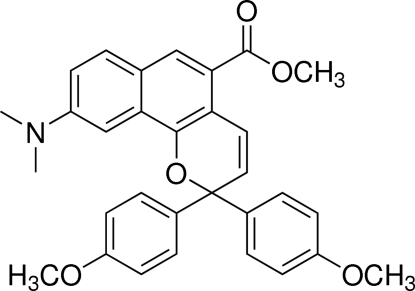


## Experimental

### 

#### Crystal data

C_31_H_29_NO_5_
*M* *_r_* = 495.55
Triclinic, 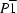

*a* = 9.8923 (1) Å
*b* = 10.9535 (1) Å
*c* = 12.1720 (2) Å
α = 93.860 (1)°
β = 112.334 (1)°
γ = 93.484 (1)°
*V* = 1211.85 (3) Å^3^
*Z* = 2
Mo *K*α radiation
μ = 0.09 mm^−1^
*T* = 100 K
0.31 × 0.20 × 0.13 mm

#### Data collection

Bruker APEXII CCD diffractometer
22031 measured reflections
6075 independent reflections
5207 reflections with *I* > 2σ(*I*)
*R*_int_ = 0.021

#### Refinement

*R*[*F* ^2^ > 2σ(*F* ^2^)] = 0.040
*wR*(*F* ^2^) = 0.111
*S* = 1.05
6075 reflections
336 parameters
H-atom parameters constrained
Δρ_max_ = 0.35 e Å^−3^
Δρ_min_ = −0.25 e Å^−3^

### 

Data collection: *APEX2* (Bruker, 2009 ▶); cell refinement: *SAINT* (Bruker, 2009 ▶); data reduction: *SAINT*; program(s) used to solve structure: *SHELXS97* (Sheldrick, 2008 ▶); program(s) used to refine structure: *SHELXL97* (Sheldrick, 2008 ▶); molecular graphics: *XP* in *SHELXTL* (Sheldrick, 2008 ▶); software used to prepare material for publication: *SHELXL97*.

## Supplementary Material

Crystal structure: contains datablocks global, I. DOI: 10.1107/S160053681101049X/pk2310sup1.cif

Structure factors: contains datablocks I. DOI: 10.1107/S160053681101049X/pk2310Isup2.hkl

Additional supplementary materials:  crystallographic information; 3D view; checkCIF report

## Figures and Tables

**Fig. 1.**
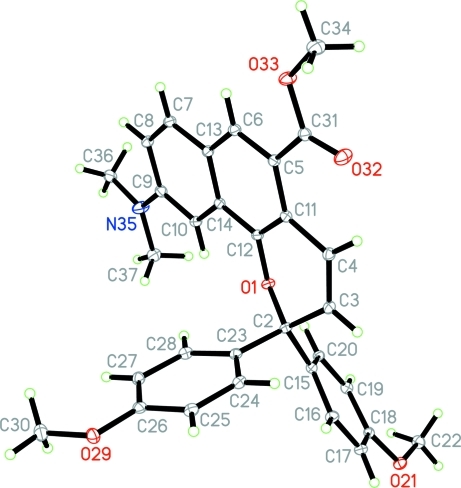
The molecular structure of the title compound with displacement ellipsoids plotted at the 30% probability level. H atoms are presented as a small spheres of arbitary radius.

**Fig. 2.**
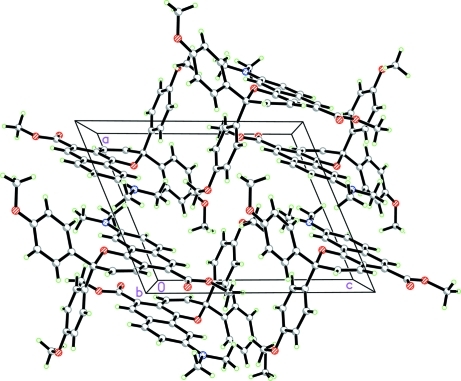
The crystal packing structure of the title compound, viewed down the b axis. [Symmetry codes: (i) x, y, z; (ii) -x, -y, -z.]

## supplementary crystallographic information

### Comment

The synthesis and application of the organic photochromic and thermochromic dyes has become of great interest recently (Kumar *et al.*, 1995; Gemert & Selvig, 2000; Nelson *et al.*, 2002; Clarke *et al.* (2002); Gabbutt *et al.*, 2003, 2004) because of potential uses as optical transmission materials, and as ophthalmic glasses and lenses. They also have potential uses in storage technologies as optical disks or memories (Crano & Guglielmetti, 1999). In particular, benzo and naphthopyrans have been commercialized as photochromic plastic sunglasses since the early 1990s. In our group, research has been focused on the development of novel photochromic benzo and naphthopyrans (Kim *et al.*, 2010; Do *et al.*, 2011). Herein, we report the crystal structure of methyl 9-(di methylamino)-2,2-bis(4-methoxyphenyl)-2*H*-benzo[*h*]chromene-5-\ carboxylate (Fig. 1 and 2) as a new photochromic material. In the title compound, C_31_H_29_NO_5_, the methyl carboxylate and the dimethylamino groups of the naphthopyran substituent are almost coplanar with the plane through the naphthopyran rings. The dihedral angle between the methyl carboxylate and the dimethylamino groups is 4.9 (1)°. The pyran ring of –C(2)—O(1)—C(12)—C(11)—C(4)—C(3)- has an envelope conformation with C(2) out of plane, C(2)—O(1) 1.448 (1) Å and C(2)—C(3) 1.512 (2) Å. The –O(21)—C(22) methoxy group of the phenyl substituent forms a dihedral angle of 16.6 (1) ° with the phenyl ring, while the –O(29)—C(30) methoxy group is almost coplanar with the plane through the phenyl ring, making a dihedral angle of 1.4 (1) °.

### Experimental

A solution of methyl 6-dimethylamino-4-hydroxy-2-naphthoate (5 mmol) and 1,1-di(4-methoxyphenyl)-prop-2-yn-1-ol (5 mmol) in toluene (60 ml) containing acidic alumina (5 g) was refluxed for 2 hrs. The cooled solution was filtered and the alumina residue washed well with ethyl acetate (3 *x* 50 ml). Removal of the dried solvent gave a yellow solid which was flash chromatographed using 20% ethyl acetate in hexane as the eluent to give an off-white solid. Single crystals of the title compound suitable for X-ray diffraction were obtained by recrystallization from ethyl acetate solution.

### Refinement

All H atoms were placed in calculated positions using a riding model, with C—H = 0.93 Å and *U*_iso_(H) = 1.2 *U*_eq_(C) for aromatic H atoms, C—H = 0.96 Å and *U*_iso_(H) = 1.5 *U*_eq_(C) for methyl H atoms.

### Crystal data

**Table:**

C_31_H_29_NO_5_	*Z* = 2
*M**_r_* = 495.55	*F*(000) = 524
Triclinic, *P*1	*D*_x_ = 1.358 Mg m^−^^3^
Hall symbol: -P 1	Mo *K*α radiation, λ = 0.71073 Å
*a* = 9.8923 (1) Å	Cell parameters from 9961 reflections
*b* = 10.9535 (1) Å	θ = 2.2–28.4°
*c* = 12.1720 (2) Å	µ = 0.09 mm^−^^1^
α = 93.860 (1)°	*T* = 100 K
β = 112.334 (1)°	Block, pale yellow
γ = 93.484 (1)°	0.31 × 0.20 × 0.13 mm
*V* = 1211.85 (3) Å^3^	

### Data collection

**Table:**

Bruker APEXII CCD diffractometer	5207 reflections with *I* > 2σ(*I*)
Radiation source: fine-focus sealed tube	*R*_int_ = 0.021
graphite	θ_max_ = 28.4°, θ_min_ = 1.8°
φ and ω scans	*h* = −13→13
22031 measured reflections	*k* = −12→14
6075 independent reflections	*l* = −16→16

### Refinement

**Table:**

Refinement on *F*^2^	Primary atom site location: structure-invariant direct methods
Least-squares matrix: full	Secondary atom site location: difference Fourier map
*R*[*F*^2^ > 2σ(*F*^2^)] = 0.040	Hydrogen site location: inferred from neighbouring sites
*wR*(*F*^2^) = 0.111	H-atom parameters constrained
*S* = 1.05	*w* = 1/[σ^2^(*F*_o_^2^) + (0.0551*P*)^2^ + 0.4305*P*] where *P* = (*F*_o_^2^ + 2*F*_c_^2^)/3
6075 reflections	(Δ/σ)_max_ < 0.001
336 parameters	Δρ_max_ = 0.35 e Å^−^^3^
0 restraints	Δρ_min_ = −0.25 e Å^−^^3^

### Special details

**Table:**

Geometry. All e.s.d.'s (except the e.s.d. in the dihedral angle between two l.s. planes) are estimated using the full covariance matrix. The cell e.s.d.'s are taken into account individually in the estimation of e.s.d.'s in distances, angles and torsion angles; correlations between e.s.d.'s in cell parameters are only used when they are defined by crystal symmetry. An approximate (isotropic) treatment of cell e.s.d.'s is used for estimating e.s.d.'s involving l.s. planes.
Refinement. Refinement of *F*^2^ against ALL reflections. The weighted *R*-factor *wR* and goodness of fit *S* are based on *F*^2^, conventional *R*-factors *R* are based on *F*, with *F* set to zero for negative *F*^2^. The threshold expression of *F*^2^ > σ(*F*^2^) is used only for calculating *R*-factors(gt) *etc*. and is not relevant to the choice of reflections for refinement. *R*-factors based on *F*^2^ are statistically about twice as large as those based on *F*, and *R*- factors based on ALL data will be even larger.

### Fractional atomic coordinates and isotropic or equivalent isotropic displacement parameters (Å^2^)

**Table:**

	*x*	*y*	*z*	*U*_iso_*/*U*_eq_	
O1	0.76711 (8)	0.30077 (7)	0.13618 (7)	0.01516 (16)	
C2	0.85589 (11)	0.20946 (10)	0.20221 (9)	0.0146 (2)	
C3	0.87855 (11)	0.11475 (10)	0.11607 (10)	0.0160 (2)	
H3A	0.8917	0.0346	0.1359	0.019*	
C4	0.87987 (12)	0.14469 (10)	0.01194 (10)	0.0166 (2)	
H4A	0.8955	0.0858	−0.0396	0.020*	
C5	0.88292 (11)	0.31985 (10)	−0.11883 (9)	0.0150 (2)	
C6	0.85247 (11)	0.43872 (10)	−0.14310 (10)	0.0157 (2)	
H6A	0.8712	0.4709	−0.2052	0.019*	
C7	0.76333 (12)	0.63497 (10)	−0.10014 (10)	0.0168 (2)	
H7A	0.7844	0.6683	−0.1607	0.020*	
C8	0.70393 (12)	0.70498 (10)	−0.03624 (10)	0.0178 (2)	
H8A	0.6867	0.7854	−0.0533	0.021*	
C9	0.66750 (12)	0.65696 (10)	0.05674 (10)	0.0176 (2)	
C10	0.70099 (12)	0.53750 (10)	0.08386 (10)	0.0161 (2)	
H10A	0.6810	0.5051	0.1454	0.019*	
C11	0.85677 (11)	0.27014 (10)	−0.02134 (9)	0.0146 (2)	
C12	0.80112 (11)	0.34390 (10)	0.04496 (9)	0.0144 (2)	
C13	0.79380 (11)	0.51228 (10)	−0.07627 (9)	0.0150 (2)	
C14	0.76453 (11)	0.46558 (10)	0.01930 (9)	0.0143 (2)	
C15	0.76705 (11)	0.14752 (10)	0.26565 (9)	0.0146 (2)	
C16	0.82776 (12)	0.05611 (10)	0.33973 (10)	0.0171 (2)	
H16A	0.9253	0.0419	0.3577	0.020*	
C17	0.74499 (12)	−0.01354 (10)	0.38671 (10)	0.0169 (2)	
H17A	0.7872	−0.0735	0.4363	0.020*	
C18	0.59832 (12)	0.00633 (10)	0.35958 (9)	0.0152 (2)	
C19	0.53879 (12)	0.10179 (10)	0.29232 (10)	0.0167 (2)	
H19A	0.4431	0.1191	0.2783	0.020*	
C20	0.62394 (12)	0.17146 (10)	0.24595 (9)	0.0158 (2)	
H20A	0.5839	0.2352	0.2009	0.019*	
O21	0.52207 (8)	−0.07485 (7)	0.40074 (7)	0.01794 (17)	
C22	0.36555 (12)	−0.07890 (11)	0.34952 (11)	0.0211 (2)	
H22A	0.3245	−0.1387	0.3851	0.032*	
H22B	0.3311	−0.1011	0.2651	0.032*	
H22C	0.3360	0.0005	0.3640	0.032*	
C23	1.00119 (11)	0.27358 (10)	0.29325 (9)	0.0149 (2)	
C24	1.12940 (12)	0.21328 (10)	0.33087 (10)	0.0170 (2)	
H24A	1.1278	0.1341	0.2973	0.020*	
C25	1.25908 (12)	0.26982 (10)	0.41753 (10)	0.0181 (2)	
H25A	1.3431	0.2281	0.4424	0.022*	
C26	1.26337 (12)	0.38915 (10)	0.46725 (10)	0.0171 (2)	
C27	1.13676 (12)	0.45073 (10)	0.43051 (10)	0.0182 (2)	
H27A	1.1387	0.5303	0.4633	0.022*	
C28	1.00713 (12)	0.39214 (10)	0.34431 (10)	0.0174 (2)	
H28A	0.9227	0.4334	0.3204	0.021*	
O29	1.39640 (9)	0.43746 (8)	0.55056 (7)	0.02068 (18)	
C30	1.40552 (14)	0.56030 (12)	0.60149 (12)	0.0259 (3)	
H30A	1.5036	0.5838	0.6583	0.039*	
H30B	1.3381	0.5650	0.6409	0.039*	
H30C	1.3810	0.6148	0.5396	0.039*	
C31	0.94328 (11)	0.24645 (10)	−0.19365 (10)	0.0161 (2)	
O32	0.97530 (10)	0.14182 (8)	−0.18347 (8)	0.0256 (2)	
O33	0.95645 (10)	0.30908 (8)	−0.28129 (7)	0.02223 (19)	
C34	1.01776 (14)	0.24406 (12)	−0.35540 (11)	0.0252 (3)	
H34A	1.0232	0.2948	−0.4152	0.038*	
H34B	0.9565	0.1696	−0.3933	0.038*	
H34C	1.1145	0.2244	−0.3071	0.038*	
N35	0.60408 (13)	0.72739 (10)	0.11748 (10)	0.0270 (2)	
C36	0.55830 (14)	0.84722 (11)	0.08327 (11)	0.0223 (2)	
H36A	0.6429	0.9023	0.0950	0.033*	
H36B	0.5084	0.8789	0.1315	0.033*	
H36C	0.4933	0.8398	0.0007	0.033*	
C37	0.56895 (14)	0.67773 (11)	0.21202 (11)	0.0241 (3)	
H37A	0.5018	0.6052	0.1801	0.036*	
H37B	0.5246	0.7379	0.2450	0.036*	
H37C	0.6572	0.6573	0.2734	0.036*	

### Atomic displacement parameters (Å^2^)

**Table:**

	*U*^11^	*U*^22^	*U*^33^	*U*^12^	*U*^13^	*U*^23^
O1	0.0179 (4)	0.0152 (4)	0.0146 (4)	0.0045 (3)	0.0076 (3)	0.0057 (3)
C2	0.0161 (5)	0.0134 (5)	0.0146 (5)	0.0033 (4)	0.0056 (4)	0.0048 (4)
C3	0.0178 (5)	0.0129 (5)	0.0180 (5)	0.0029 (4)	0.0072 (4)	0.0029 (4)
C4	0.0177 (5)	0.0147 (5)	0.0175 (5)	0.0017 (4)	0.0070 (4)	0.0010 (4)
C5	0.0150 (5)	0.0154 (5)	0.0142 (5)	0.0003 (4)	0.0055 (4)	0.0013 (4)
C6	0.0149 (5)	0.0174 (5)	0.0148 (5)	0.0003 (4)	0.0056 (4)	0.0033 (4)
C7	0.0170 (5)	0.0176 (5)	0.0156 (5)	0.0007 (4)	0.0055 (4)	0.0045 (4)
C8	0.0196 (5)	0.0148 (5)	0.0178 (5)	0.0023 (4)	0.0055 (4)	0.0040 (4)
C9	0.0193 (5)	0.0170 (5)	0.0157 (5)	0.0025 (4)	0.0058 (4)	0.0006 (4)
C10	0.0181 (5)	0.0164 (5)	0.0140 (5)	0.0019 (4)	0.0061 (4)	0.0029 (4)
C11	0.0146 (4)	0.0144 (5)	0.0135 (5)	0.0006 (4)	0.0042 (4)	0.0016 (4)
C12	0.0140 (4)	0.0159 (5)	0.0126 (5)	0.0002 (4)	0.0044 (4)	0.0032 (4)
C13	0.0141 (4)	0.0153 (5)	0.0140 (5)	−0.0001 (4)	0.0039 (4)	0.0025 (4)
C14	0.0135 (4)	0.0141 (5)	0.0134 (5)	0.0003 (4)	0.0032 (4)	0.0020 (4)
C15	0.0169 (5)	0.0137 (5)	0.0129 (5)	0.0005 (4)	0.0057 (4)	0.0008 (4)
C16	0.0152 (5)	0.0188 (5)	0.0176 (5)	0.0025 (4)	0.0062 (4)	0.0040 (4)
C17	0.0187 (5)	0.0170 (5)	0.0155 (5)	0.0033 (4)	0.0063 (4)	0.0050 (4)
C18	0.0183 (5)	0.0148 (5)	0.0131 (5)	−0.0006 (4)	0.0073 (4)	−0.0002 (4)
C19	0.0163 (5)	0.0175 (5)	0.0170 (5)	0.0033 (4)	0.0072 (4)	0.0011 (4)
C20	0.0186 (5)	0.0140 (5)	0.0143 (5)	0.0030 (4)	0.0055 (4)	0.0017 (4)
O21	0.0169 (4)	0.0192 (4)	0.0202 (4)	0.0012 (3)	0.0094 (3)	0.0051 (3)
C22	0.0169 (5)	0.0254 (6)	0.0205 (5)	−0.0022 (4)	0.0075 (4)	0.0019 (5)
C23	0.0165 (5)	0.0161 (5)	0.0138 (5)	0.0011 (4)	0.0075 (4)	0.0038 (4)
C24	0.0190 (5)	0.0149 (5)	0.0187 (5)	0.0024 (4)	0.0086 (4)	0.0036 (4)
C25	0.0168 (5)	0.0192 (5)	0.0196 (5)	0.0035 (4)	0.0075 (4)	0.0059 (4)
C26	0.0177 (5)	0.0200 (5)	0.0144 (5)	−0.0014 (4)	0.0071 (4)	0.0043 (4)
C27	0.0223 (5)	0.0159 (5)	0.0175 (5)	0.0014 (4)	0.0090 (4)	0.0013 (4)
C28	0.0183 (5)	0.0177 (5)	0.0180 (5)	0.0038 (4)	0.0086 (4)	0.0039 (4)
O29	0.0180 (4)	0.0219 (4)	0.0193 (4)	−0.0022 (3)	0.0050 (3)	0.0003 (3)
C30	0.0250 (6)	0.0247 (6)	0.0251 (6)	−0.0044 (5)	0.0085 (5)	−0.0036 (5)
C31	0.0145 (5)	0.0175 (5)	0.0153 (5)	0.0001 (4)	0.0049 (4)	0.0019 (4)
O32	0.0370 (5)	0.0195 (4)	0.0298 (5)	0.0100 (4)	0.0215 (4)	0.0072 (4)
O33	0.0324 (4)	0.0210 (4)	0.0216 (4)	0.0084 (3)	0.0182 (4)	0.0059 (3)
C34	0.0340 (6)	0.0259 (6)	0.0248 (6)	0.0081 (5)	0.0203 (5)	0.0042 (5)
N35	0.0446 (6)	0.0205 (5)	0.0262 (5)	0.0144 (5)	0.0224 (5)	0.0080 (4)
C36	0.0291 (6)	0.0173 (5)	0.0191 (6)	0.0073 (4)	0.0074 (5)	0.0001 (4)
C37	0.0332 (6)	0.0211 (6)	0.0237 (6)	0.0063 (5)	0.0167 (5)	0.0022 (5)

### Geometric parameters (Å, °)

**Table:**

O1—C12	1.3800 (12)	C19—H19A	0.9300
O1—C2	1.4478 (12)	C20—H20A	0.9300
C2—C3	1.5120 (15)	O21—C22	1.4291 (13)
C2—C15	1.5306 (14)	C22—H22A	0.9600
C2—C23	1.5327 (14)	C22—H22B	0.9600
C3—C4	1.3350 (15)	C22—H22C	0.9600
C3—H3A	0.9300	C23—C28	1.3894 (15)
C4—C11	1.4654 (15)	C23—C24	1.3984 (15)
C4—H4A	0.9300	C24—C25	1.3889 (15)
C5—C6	1.3802 (15)	C24—H24A	0.9300
C5—C11	1.4404 (14)	C25—C26	1.3942 (16)
C5—C31	1.4851 (15)	C25—H25A	0.9300
C6—C13	1.4054 (15)	C26—O29	1.3671 (13)
C6—H6A	0.9300	C26—C27	1.3933 (15)
C7—C8	1.3638 (16)	C27—C28	1.3943 (16)
C7—C13	1.4200 (15)	C27—H27A	0.9300
C7—H7A	0.9300	C28—H28A	0.9300
C8—C9	1.4333 (15)	O29—C30	1.4260 (15)
C8—H8A	0.9300	C30—H30A	0.9600
C9—N35	1.3675 (15)	C30—H30B	0.9600
C9—C10	1.3995 (15)	C30—H30C	0.9600
C10—C14	1.4090 (15)	C31—O32	1.2094 (14)
C10—H10A	0.9300	C31—O33	1.3486 (13)
C11—C12	1.3824 (15)	O33—C34	1.4390 (14)
C12—C14	1.4261 (14)	C34—H34A	0.9600
C13—C14	1.4202 (14)	C34—H34B	0.9600
C15—C20	1.3879 (15)	C34—H34C	0.9600
C15—C16	1.3998 (15)	N35—C36	1.4504 (15)
C16—C17	1.3849 (15)	N35—C37	1.4513 (15)
C16—H16A	0.9300	C36—H36A	0.9600
C17—C18	1.3948 (15)	C36—H36B	0.9600
C17—H17A	0.9300	C36—H36C	0.9600
C18—O21	1.3698 (13)	C37—H37A	0.9600
C18—C19	1.3908 (15)	C37—H37B	0.9600
C19—C20	1.3970 (15)	C37—H37C	0.9600
			
C12—O1—C2	116.99 (8)	C15—C20—H20A	119.3
O1—C2—C3	109.42 (8)	C19—C20—H20A	119.3
O1—C2—C15	105.97 (8)	C18—O21—C22	117.02 (8)
C3—C2—C15	109.14 (8)	O21—C22—H22A	109.5
O1—C2—C23	109.16 (8)	O21—C22—H22B	109.5
C3—C2—C23	112.37 (9)	H22A—C22—H22B	109.5
C15—C2—C23	110.58 (8)	O21—C22—H22C	109.5
C4—C3—C2	120.87 (10)	H22A—C22—H22C	109.5
C4—C3—H3A	119.6	H22B—C22—H22C	109.5
C2—C3—H3A	119.6	C28—C23—C24	118.21 (10)
C3—C4—C11	120.16 (10)	C28—C23—C2	120.43 (9)
C3—C4—H4A	119.9	C24—C23—C2	121.30 (10)
C11—C4—H4A	119.9	C25—C24—C23	121.02 (10)
C6—C5—C11	119.51 (10)	C25—C24—H24A	119.5
C6—C5—C31	118.98 (10)	C23—C24—H24A	119.5
C11—C5—C31	121.51 (9)	C24—C25—C26	120.02 (10)
C5—C6—C13	121.62 (10)	C24—C25—H25A	120.0
C5—C6—H6A	119.2	C26—C25—H25A	120.0
C13—C6—H6A	119.2	O29—C26—C27	124.62 (10)
C8—C7—C13	121.52 (10)	O29—C26—C25	115.66 (10)
C8—C7—H7A	119.2	C27—C26—C25	119.71 (10)
C13—C7—H7A	119.2	C26—C27—C28	119.52 (10)
C7—C8—C9	121.28 (10)	C26—C27—H27A	120.2
C7—C8—H8A	119.4	C28—C27—H27A	120.2
C9—C8—H8A	119.4	C23—C28—C27	121.51 (10)
N35—C9—C10	121.68 (10)	C23—C28—H28A	119.2
N35—C9—C8	120.42 (10)	C27—C28—H28A	119.2
C10—C9—C8	117.90 (10)	C26—O29—C30	117.17 (9)
C9—C10—C14	120.94 (10)	O29—C30—H30A	109.5
C9—C10—H10A	119.5	O29—C30—H30B	109.5
C14—C10—H10A	119.5	H30A—C30—H30B	109.5
C12—C11—C5	118.40 (10)	O29—C30—H30C	109.5
C12—C11—C4	116.34 (9)	H30A—C30—H30C	109.5
C5—C11—C4	125.22 (10)	H30B—C30—H30C	109.5
O1—C12—C11	121.65 (9)	O32—C31—O33	121.29 (10)
O1—C12—C14	115.09 (9)	O32—C31—C5	126.67 (10)
C11—C12—C14	123.10 (10)	O33—C31—C5	112.01 (9)
C6—C13—C7	121.91 (10)	C31—O33—C34	114.80 (9)
C6—C13—C14	120.37 (10)	O33—C34—H34A	109.5
C7—C13—C14	117.72 (10)	O33—C34—H34B	109.5
C10—C14—C13	120.55 (10)	H34A—C34—H34B	109.5
C10—C14—C12	122.51 (10)	O33—C34—H34C	109.5
C13—C14—C12	116.94 (10)	H34A—C34—H34C	109.5
C20—C15—C16	118.16 (10)	H34B—C34—H34C	109.5
C20—C15—C2	122.70 (9)	C9—N35—C36	121.78 (10)
C16—C15—C2	118.96 (9)	C9—N35—C37	119.72 (10)
C17—C16—C15	121.08 (10)	C36—N35—C37	118.27 (10)
C17—C16—H16A	119.5	N35—C36—H36A	109.5
C15—C16—H16A	119.5	N35—C36—H36B	109.5
C16—C17—C18	119.93 (10)	H36A—C36—H36B	109.5
C16—C17—H17A	120.0	N35—C36—H36C	109.5
C18—C17—H17A	120.0	H36A—C36—H36C	109.5
O21—C18—C19	124.78 (10)	H36B—C36—H36C	109.5
O21—C18—C17	115.47 (9)	N35—C37—H37A	109.5
C19—C18—C17	119.74 (10)	N35—C37—H37B	109.5
C18—C19—C20	119.46 (10)	H37A—C37—H37B	109.5
C18—C19—H19A	120.3	N35—C37—H37C	109.5
C20—C19—H19A	120.3	H37A—C37—H37C	109.5
C15—C20—C19	121.37 (10)	H37B—C37—H37C	109.5
			
C12—O1—C2—C3	−43.91 (11)	O1—C2—C15—C16	−178.39 (9)
C12—O1—C2—C15	−161.45 (8)	C3—C2—C15—C16	63.88 (12)
C12—O1—C2—C23	79.44 (11)	C23—C2—C15—C16	−60.22 (13)
O1—C2—C3—C4	29.20 (13)	C20—C15—C16—C17	3.65 (16)
C15—C2—C3—C4	144.74 (10)	C2—C15—C16—C17	−171.58 (10)
C23—C2—C3—C4	−92.22 (12)	C15—C16—C17—C18	0.51 (17)
C2—C3—C4—C11	−1.02 (16)	C16—C17—C18—O21	173.93 (10)
C11—C5—C6—C13	−1.19 (16)	C16—C17—C18—C19	−4.52 (16)
C31—C5—C6—C13	179.32 (9)	O21—C18—C19—C20	−174.01 (10)
C13—C7—C8—C9	−0.99 (17)	C17—C18—C19—C20	4.28 (16)
C7—C8—C9—N35	−178.09 (11)	C16—C15—C20—C19	−3.87 (16)
C7—C8—C9—C10	2.88 (16)	C2—C15—C20—C19	171.16 (10)
N35—C9—C10—C14	179.14 (10)	C18—C19—C20—C15	−0.05 (16)
C8—C9—C10—C14	−1.84 (16)	C19—C18—O21—C22	14.49 (15)
C6—C5—C11—C12	0.19 (15)	C17—C18—O21—C22	−163.87 (10)
C31—C5—C11—C12	179.67 (9)	O1—C2—C23—C28	31.18 (13)
C6—C5—C11—C4	178.05 (10)	C3—C2—C23—C28	152.75 (10)
C31—C5—C11—C4	−2.48 (16)	C15—C2—C23—C28	−85.02 (12)
C3—C4—C11—C12	−14.54 (15)	O1—C2—C23—C24	−151.49 (9)
C3—C4—C11—C5	167.56 (10)	C3—C2—C23—C24	−29.92 (13)
C2—O1—C12—C11	32.06 (13)	C15—C2—C23—C24	92.30 (12)
C2—O1—C12—C14	−152.36 (9)	C28—C23—C24—C25	0.44 (16)
C5—C11—C12—O1	177.05 (9)	C2—C23—C24—C25	−176.94 (10)
C4—C11—C12—O1	−1.00 (15)	C23—C24—C25—C26	−0.89 (16)
C5—C11—C12—C14	1.82 (15)	C24—C25—C26—O29	−179.12 (9)
C4—C11—C12—C14	−176.23 (9)	C24—C25—C26—C27	0.66 (16)
C5—C6—C13—C7	179.48 (10)	O29—C26—C27—C28	179.75 (10)
C5—C6—C13—C14	0.22 (16)	C25—C26—C27—C28	−0.01 (16)
C8—C7—C13—C6	178.80 (10)	C24—C23—C28—C27	0.23 (16)
C8—C7—C13—C14	−1.91 (16)	C2—C23—C28—C27	177.64 (10)
C9—C10—C14—C13	−1.04 (16)	C26—C27—C28—C23	−0.45 (17)
C9—C10—C14—C12	179.53 (10)	C27—C26—O29—C30	−1.02 (16)
C6—C13—C14—C10	−177.79 (9)	C25—C26—O29—C30	178.75 (10)
C7—C13—C14—C10	2.91 (15)	C6—C5—C31—O32	179.97 (11)
C6—C13—C14—C12	1.67 (15)	C11—C5—C31—O32	0.49 (17)
C7—C13—C14—C12	−177.63 (9)	C6—C5—C31—O33	−1.79 (14)
O1—C12—C14—C10	1.21 (15)	C11—C5—C31—O33	178.72 (9)
C11—C12—C14—C10	176.72 (10)	O32—C31—O33—C34	−3.09 (16)
O1—C12—C14—C13	−178.24 (8)	C5—C31—O33—C34	178.57 (9)
C11—C12—C14—C13	−2.73 (15)	C10—C9—N35—C36	−174.87 (11)
O1—C2—C15—C20	6.61 (14)	C8—C9—N35—C36	6.14 (18)
C3—C2—C15—C20	−111.12 (11)	C10—C9—N35—C37	−0.52 (18)
C23—C2—C15—C20	124.78 (11)	C8—C9—N35—C37	−179.51 (11)

### Hydrogen-bond geometry (Å, °)

**Table:**

*D*—H···*A*	*D*—H	H···*A*	*D*···*A*	*D*—H···*A*
?—?···?	?	?	?	?
